# How the digital literacy level of teachers affects student well-being: a configuration analysis approach based on fsQCA

**DOI:** 10.3389/fpubh.2026.1779421

**Published:** 2026-03-26

**Authors:** Liyan Chen, Yuanyuan Qiu, Bo Zhong

**Affiliations:** 1 Wenzhou Business College, Wenzhou, China; 2 Zhejiang University of Finance and Economics Dongfang College, Haining, China; 3 Nanjing University, Nanjing, China

**Keywords:** configuration analysis method, digital literacy, fsQCA, student well-being, teacher

## Abstract

As digital education continues to deepen, how teachers’ digital literacy affects students’ well-being has become an important issue with both educational value and public health significance. To move beyond the limitations of traditional variable-centered thinking and to respond to the theoretical demand of viewing digital literacy as a complex system, this study adopts a configurational theoretical perspective and employs fuzzy-set qualitative comparative analysis (fsQCA). Based on data from 540 questionnaire surveys, it systematically explores the multiple concurrent causal mechanisms through which different dimensions of teachers’ digital literacy influence students’ well-being. The findings show that: (1) professional development, digital application, and digital social responsibility are core necessary conditions for enhancing students’ well-being; (2) digital awareness and digital technology knowledge and skills, as auxiliary conditions, form multiple equivalent configurational pathways in combination with the core conditions, indicating that there is no single optimal path to improving students’ well-being; and (3) the multidimensional synergistic effects of teachers’ digital literacy highlight the importance of systematic integration, as improvement in a single dimension alone is insufficient to comprehensively promote students’ well-being. This study not only methodologically demonstrates the applicability of configurational analysis in revealing complex education–health mechanisms, but also extends the theoretical explanation of adolescent well-being as a public health outcome from a systems perspective. It provides evidence-based intervention implications for teacher development in the digital transformation of education, thereby linking the dual goals of improving educational quality and promoting population health at both theoretical and practical levels.

## 1 Introduction

Student mental health and well-being, recognized as a critical public health issue, have gained global consensus on their importance. Large-scale studies, such as analyses of PISA (Program for International Student Assessment) data, not only reveal the significant impact of contextual factors like school climate and teacher-student relationships on students’ life satisfaction and mental health (1) but also track the vulnerability and group differences in students’ psychological well-being under external shocks like the COVID-19 pandemic (2). This underscores the necessity of monitoring and intervening at the population health level. In this context, the education system, due to its universality and accessibility, is regarded as a core intervention setting for promoting adolescent mental health.

Building on PISA’s student well-being measurement scales, this study deeply understands that, from a public health and population health perspective, student well-being is a multidimensional concept with significant public health implications. The World Health Organization, in its Global School Health Initiative, clearly states that student well-being encompasses life satisfaction, positive school experiences, mental health, supportive social relationships, and personal development potential. Therefore, PISA research not only emphasizes that well-being is a combination of positive emotions and optimistic attitudes toward the future but also further reveals its population distribution characteristics and social determinants, elevating individual-level feelings into the measurable and intervenable domain of public health. Through assessment tools like PISA, this study recognizes that focusing on students’ collective well-being is a crucial indicator for evaluating the health outcomes of the education system, optimizing policies and environments, and thereby promoting students’ holistic development and long-term well-being. This comprehensive perspective, integrating educational science and public health, prompts a rethinking of educational goals and methods, ensuring that every student can grow healthily in a supportive environment while pursuing academic achievements, laying a solid foundation for their personal and societal better future. Specifically, individual well-being focuses on students’ subjective experiences and functional states, while well-being at the public health level concentrates on the health distribution of student populations, related risk factors, and preventable health burdens. The two are closely linked because individual well-being forms the basis of population health, and population-level policies, environments, and social support profoundly influence the realization of each student’s well-being. Systematically incorporating student well-being into the public health domain is theoretically grounded in three aspects: first, students are a large-scale population group in a critical developmental period, and their overall well-being is central to future societal health capital; second, early investment in psychosocial well-being can effectively prevent physical and mental illnesses in adulthood, offering high public health benefits; and finally, focusing on and reducing disparities in well-being within student populations is a key entry point for advancing health equity.

In this context, teachers play a pivotal role. Existing research suggests that the synergistic integration of external academic support and personal internal resources is key to sustaining student well-being (3), and structured positive education programs can effectively enhance students’ mental health (4). However, as the digital transformation of education accelerates, traditional support mechanisms and teacher-student interactions are being reshaped. Teachers’ digital literacy, as the core of teachers’ professional competence in the digital era, has become a concrete vehicle for shaping the digital school climate and providing digital academic support (5–7). Studies indicate that teachers’ digital literacy not only influences their curriculum leadership and the cultivation of online classroom environments (8) but also that their technological confidence can reduce instructional disruptions and teacher-student anxiety (9). Therefore, investigating how teachers’ digital literacy influences student well-being is an urgent issue for integrating public health goals into digital education practices.

Although enhancing teachers’ digital literacy is a policy consensus, with related frameworks and training programs continually emerging, existing research exhibits significant theoretical limitations and perspective gaps in explaining its relationship with student well-being (10–12). Firstly, from a theoretical perspective, most studies follow a variable-centric approach, examining the independent or net effects of various dimensions of digital literacy [e.g., technological knowledge, school support; (13)]. This method fails to uncover potential asymmetric, synergistic, or substitutive configurational relationships among dimensions, overlooking the fact that as a complex system, the impact of digital literacy may stem from specific combinations of elements rather than from single dimensions. Secondly, from a research perspective, existing literature predominantly focuses on the impact of digital literacy on teaching efficiency or academic achievement, failing to explicitly and systematically examine student well-being as a core health outcome variable from a public health perspective. This disconnect has resulted in a research “black box” (14): it remains unclear which specific combinations of teachers’ digital literacy, through what concurrent causal pathways, ultimately enhance student well-being as a public health asset.

To open this “black box,” this study, based on the aforementioned theoretical and practical background, introduces a configurational theory perspective and employs the fuzzy-set Qualitative Comparative Analysis (fsQCA) method. The aim is to explore how various dimensions of teachers’ digital literacy (referencing the Chinese Ministry of Education’s *Teachers’ Digital Literacy* standard) combine in different forms to constitute sufficient conditional pathways leading to high student well-being. Building on a survey of 540 teachers in the Chinese context and synthesizing existing research findings and theoretical models, this study seeks to answer: whether there exist multiple configurations of digital literacy that enhance student well-being; whether there are core conditions with universal necessity; and the mechanisms through which teachers’ digital literacy operates in the formation of student well-being. By uncovering these complex causal mechanisms, this study not only aims to deepen the theoretical understanding of teachers’ digital literacy as a synergistic system, addressing the shortcomings of the existing variable-centric paradigm, and thereby deriving insights for improving teaching quality and enhancing student well-being in the context of digital transformation in education. More importantly, from the intersection of educational policy and public health practice, it seeks to provide solid empirical evidence and precise intervention insights for designing systematic strategies for developing teachers’ digital literacy with the explicit goal of promoting adolescent mental health.

## 2 Theoretical background and model construction

### 2.1 Teachers’ digital literacy

This study adopts Urie Bronfenbrenner’s ecological systems theory as a macro framework to explore how teachers’ digital literacy influences student well-being (15). The theory posits that individual development is nested within a series of interconnected environmental systems, with the microsystem—where individuals directly experience face-to-face interactions—having the most immediate impact. From a public health perspective, the school environment serves as a core intervention site for promoting adolescent mental health (16). Within this school environment, digitally reshaped classrooms and learning spaces constitute a crucial digital microsystem for student development (17). Teachers, as the primary constructors and agents within this microsystem, directly determine its quality, atmosphere, and supportiveness through their digital literacy. Teacher digital literacy refers to the awareness, competence, and responsibility of teachers to appropriately utilize digital technology to acquire, process, use, manage, and evaluate digital information and resources, to identify, analyze, and solve educational problems, and to optimize, innovate, and transform educational activities. In the present era, the level of teacher digital literacy has become a core professional competence for educators (18, 19). Therefore, drawing upon the “Teacher Digital Literacy” standard researched and formulated by China’s Ministry of Education in 2022, this study conceptualizes teacher digital literacy as a key set of competencies enabling teachers to construct a positive, safe, and effective digital learning microsystem. The five dimensions—digital awareness, digital knowledge and skills, digital application, digital social responsibility, and professional development—do not operate independently on students. Instead, they combine to form different systemic attributes, acting as “core conditions” or “supporting conditions” that interact synergistically with other contextual factors to holistically shape the student experience and environment, thereby influencing their psychological well-being.

#### 2.1.1 Digital awareness: driving and guiding dimensions

Digital awareness refers to teachers’ understanding of the value of digital technology in socioeconomic and educational development, as well as their attitudes toward digital technology resources and their application in teaching and learning. It encompasses digital recognition, digital willingness, and digital resolve. As a driving condition within the configuration, it determines teachers’ initiative in exploring and integrating other dimensions of digital literacy. High levels of digital recognition enable teachers to understand how digital tools can optimize educational processes and provide more diverse learning experiences. This not only enhances students’ sense of engagement and learning interest but also helps them better prepare for future societal challenges (20, 21). Teachers’ digital willingness ensures they continually explore and apply the latest educational technologies to create an innovative and stimulating learning environment for students. Teachers’ digital resolve empowers them to understand the process through which digital tools optimize education, allowing them to provide more varied learning experiences and thereby persistently drive the optimization of the teaching environment (22). This actively created, engaging learning environment can stimulate students’ creativity and thirst for knowledge, enhance their educational experience, and thus promote their personal growth and development (23, 24). This provides an important contextual foundation for students to increase their sense of participation, enhance their learning interest and psychological well-being (20) and also exerts a significant influence on students’ psychological happiness and overall well-being. Therefore, digital awareness constitutes the fundamental internal motivation for teachers to proactively optimize the digital microsystem they construct.

#### 2.1.2 Digital technology knowledge and skills: basic tool dimensions

Digital technology knowledge and skills refer to the knowledge of digital technologies that teachers should understand and the digital technology skills they need to master in their daily educational and teaching activities. This dimension measures teachers’ ability to use specific digital tools, serving as a foundational condition for configuration and providing tool support for other dimensions, especially the “digital application” dimension. When teachers possess solid digital technology knowledge—such as understanding concepts and basic principles of cloud computing, big data, artificial intelligence, and more—they can design teaching content more effectively and use digital tools to provide more engaging learning experiences (25, 26). This modern teaching approach not only enhances students’ interest in learning but also improves their learning efficiency, leading to a sense of satisfaction in their learning outcomes. The impact of digital literacy and resource quality on teaching outcomes is extensive, with research findings indicating that proficient use of digital tools like artificial intelligence can not only enhance interactivity and student engagement but also address challenges arising from the digital divide and unequal access to technological resources (27). At the same time, exploring ways to improve teachers’ digital literacy levels also contributes to understanding the digital transformation in higher education and provides insights into cultivating teacher competencies that meet the demands of modern education (28). Teachers’ digital technology skills, such as proficiency in using online learning platforms, multimedia tools, and data analysis software, can help them better personalize teaching and reduce learning barriers in the era of big data (29). These skills can directly meet students’ need for a sense of competence and enhance their self-confidence, serving as a key element in creating a supportive learning environment. These skills not only improve students’ academic achievements but also significantly enhance their overall well-being throughout their student careers. This knowledge and these skills form the instrumental foundation necessary for teachers to effectively establish and operate digital microsystems.

#### 2.1.3 Digital application: teaching practice dimension

This dimension refers to teachers’ ability to apply digital technology resources in educational and teaching activities, including their capacity to integrate digital technology into the entire teaching process—such as instructional design, implementation, evaluation, and collaborative education. It serves as the practical application of digital literacy, directly shaping the quality of classroom interactions and learning experiences. Often, it works in conjunction with other dimensions, such as “knowledge and skills” and “social responsibility,” to form specific teaching practice models. Through digital instructional design, teachers can more accurately analyze students’ learning situations and tailor personalized learning plans, enabling students to improve their learning abilities in a mode that suits them best. During the implementation of digital teaching, teachers use digital tools to optimize teaching processes, increase classroom interaction, and enhance student engagement, allowing students to gain a sense of achievement in a positive learning atmosphere. Digital academic evaluation, through data-driven approaches, enables students to clearly see their growth and shortcomings, stimulating intrinsic motivation rather than relying solely on external grading (30). Additionally, teachers’ digital collaborative education abilities—such as communicating with parents through digital platforms to jointly focus on students’ mental health and holistic development—create a more supportive and understanding learning and living environment for students, thereby enhancing their sense of happiness and security. Therefore, digital application is the core practical process through which teachers operate and interact with the digital microsystems they construct, directly determining students’ immediate experiences within the system.

#### 2.1.4 . Digital social responsibility: ethics and security protection dimension

This dimension emphasizes the responsibilities of teachers in terms of ethical cultivation and behavioral norms in digital activities, including legal and ethical standards as well as digital security protection. It plays the role of a safeguarding condition in the configuration. In the school environment, teachers in the digital age have the responsibility to provide information and network education in addition to imparting knowledge. Guiding students to understand personal information security knowledge and privacy rights is particularly important in the digital era. In the internet age, adolescents are exposed to aggressive content, threats, and privacy violations related to digital technology, which may negatively affect their mental health (31). Therefore, developing digitally literate teachers is crucial to helping them maintain well-being (32). A teacher with a strong sense of digital social responsibility can guide students to comply with relevant laws and regulations, educate them on ethical norms, thereby effectively preventing online risks and promoting the creation of a safe and trustworthy digital environment (33). This allows students to participate in online learning activities more freely and with greater peace of mind. Additionally, teachers’ emphasis on and maintenance of data security can further enhance students’ sense of trust and safety. These behaviors of teachers not only protect students’ personal rights but also reduce potential psychological pressure (28). Learning in a safe and protected environment enables students to better explore themselves and develop skills, which is crucial for their resilience building and emotional development. Such a safe environment is a necessary but insufficient condition for students to gain psychological security and reduce anxiety and stress. It must be combined with other dimensions to transform protection into a positive growth experience. In summary, digital social responsibility is the foundational safeguard ensuring that the digital microsystem constructed by teachers is safe, trustworthy, and ethically compliant, serving as a prerequisite for students to gain psychological security within it.

#### 2.1.5 Professional development: the dimension of iteration and innovation

This dimension refers to the ability of teachers to use digital technology resources to promote their own and their community’s professional development, including digital learning and training, as well as digital teaching research and innovation. It serves as an evolutionary condition in the configuration, ensuring that teachers’ digital literacy practices can continuously update and adapt. Through professional development, teachers use digital technology resources for ongoing learning and training, which not only updates and enhances their teaching skills but also introduces innovative models, providing students with richer and more efficient learning experiences and improving their professional performance (34, 35). Teachers’ digital teaching research and innovation further drive the innovation of teaching methods, enabling students to enhance their learning abilities in an environment that stimulates interest and creativity (36). This continuous learning and improvement reflects the growth mindset of educators. Teachers can not only meet the personalized needs of students and stimulate their learning interest, thereby enhancing their learning self-efficacy (37), but also set an example of lifelong learning, allowing students to find joy and satisfaction in exploration and growth, creating a positive and motivating classroom learning atmosphere. This is crucial for cultivating students’ growth mindset and intrinsic well-being. Therefore, professional development ensures that the digital microsystem constructed by teachers can continuously evolve, adapt, and innovate, thereby maintaining its supportive efficacy for student development in the long term.

In summary, from the theoretical perspective of this study, teachers’ digital literacy is defined as a multi-dimensional set of capabilities that enable teachers to construct digital learning microsystems that promote student well-being. The aforementioned five dimensions outline different construction aspects of this system, namely the driving, tool, practice, guarantee, and evolution dimensions. These dimensions do not influence students independently or in parallel but can be combined in asymmetric and diverse configurations, collectively defining various types of microsystems. The subsequent configurational analysis, guided by ecological systems theory, systematically identifies the combination patterns of teachers’ literacy conditions that most effectively shape positive digital microsystems and, in turn, foster student well-being.

### 2.2 Measuring digital literacy levels

To solidly enhance teachers’ awareness, ability, and responsibility in using digital technology to optimize, innovate, and transform educational and teaching activities, the Ministry of Education of China studied and formulated the “Teacher Digital Literacy” standard in 2022. This standard serves as the mainstream indicator for measuring the digital proficiency of teachers in China today. It constructs a teacher digital literacy evaluation system from five dimensions: Digital Awareness, Digital Technology and Skills, Digital Practical Ability, Digital Social Responsibility, and Professional Development. This framework systematically refines the core components of teacher digital literacy.

This study adopts this framework as the basis for measuring teachers’ digital literacy, primarily because of its alignment with the research questions and its cross-contextual universality and referential value, for the following reasons. First, the five-dimensional structure of the framework comprehensively covers the core elements of teachers’ digital literacy widely recognized in international educational research. It includes not only technical competencies such as mastery of digital technologies and digital teaching applications, but also non-technical dimensions such as digital awareness, digital social responsibility, and professional development. These dimensions closely align with the core requirements for teachers’ digital literacy articulated in ICT competency frameworks proposed by international organizations such as UNESCO, reflecting the shared professional competency demands placed on teachers in the digital era. Second, the framework balances practicality and generalizability in its indicator design. Its dimensional logic is not constrained by the educational systems or instructional models of any single country, providing a comparable analytical basis for exploring the composition and mechanisms of teachers’ digital literacy across different countries and regions, and demonstrating potential for cross-cultural adaptation and validation. Third, the indicators within each dimension are clearly defined and quantifiable. They are suitable not only for the empirical analysis of basic education contexts in China conducted in this study, but also offer methodological references for similar studies in other countries. Moreover, the configurational analysis logic of the framework is highly compatible with the fsQCA method adopted in this study, facilitating the examination of multidimensional combinational effects.

Based on these characteristics, this study employs the five-dimensional framework of the *Standards for Teachers’ Digital Literacy* to examine the effects of individual dimensions and their configurations of teachers’ digital literacy on students’ well-being. Detailed descriptions of the indicators are presented in Table 1.

**Table 1:** Teacher digital literacy.

First-level dimensions	Connotation
Digital awareness	Teachers’ understanding of the value of digital technology in economic, social, and educational development, as well as their attitude toward digital technology resources and their application in teaching and education
Digital technology knowledge and skills	The digital technology knowledge teachers should be aware of and the digital technology skills they need to master in daily educational and teaching activities
Digital applications	The ability of teachers to use digital technology resources to carry out educational and teaching activities, including digital instructional design, digital teaching implementation, digital academic evaluation, and digital collaborative education.
Digital social responsibility	The responsibility of teachers regarding moral cultivation and behavioral norms in digital activities, including legal and ethical standards, as well as digital security protection.
Professional development	The ability of teachers to use digital technology resources to promote their own and community’s professional development, including digital learning and research, as well as digital teaching research and innovation.

Source: Education Industry Standard of the People’s Republic of China JY/T0646-2022.

### 2.3 Student well-being

The sense of well-being among young students, as a core psychological well-being indicator of concern in the field of public health, is directly associated with their physical and mental health status, the formation of behavioral patterns, and long-term developmental potential. It has become a key research focus at the intersection of global higher education and public health (38). As a foundational psychological resource for the holistic development of adolescents, student well-being is not only an important safeguard of individual physical and mental health, but also reflects the overall level of group mental health and the public health suitability of educational environments, serving as a critical dimension for assessing the quality of health-oriented services within education systems (39).

As public health concepts extend toward a “whole-population, whole-life-course” approach, adolescent well-being, as a core health indicator in the non-cognitive domain, has seen its distributional characteristics, influencing factors, and intervention pathways receive increasing attention from public health scholars, education administrators, policymakers, and society at large worldwide. From the perspective of public health practice, constructing a scientific and systematic framework for assessing student well-being and exploring diversified assessment tools are of great significance for accurately identifying adolescent mental health risks, strengthening school belonging and self-efficacy, and intervening in negative emotions and other public health issues (26, 40). Existing public health–related research has confirmed that enhancing students’ digital literacy is not only an important measure for promoting their academic and career development, but can also exert positive effects on their mental health by improving psychological adaptability and strengthening social connectedness, thereby contributing to the comprehensive enhancement of adolescent health literacy (32). Further studies indicate that, alongside the promotion of adolescents’ reading, mathematics, and science literacy, strengthening well-being interventions, cultivating positive psychological qualities, and developing emotion regulation capacities constitute key synergistic strategies for improving the overall health level of adolescent populations (26).

The questionnaire design of this study draws on relevant indicator frameworks from the Program for International Student Assessment (PISA). As a large-scale assessment project with broad international recognition, PISA first incorporated well-being–related questions into its student questionnaire in 2015, initiating standardized measurement practices of well-being among 15-year-old adolescents. In 2018, it further introduced a dedicated student well-being questionnaire, whose assessment dimensions and instruments have undergone multiple rounds of validation and optimization, resulting in an evaluation framework that combines scientific rigor with practical applicability and provides an authoritative reference for population-based assessments of adolescent well-being in the field of public health.

### 2.4 Model construction

This model adopts the Complex Systems Theory as its core framework, aiming to reveal the internal structure and evolutionary mechanisms of teachers’ digital literacy. Complex Systems Theory emphasizes interactions, nonlinear relationships, and dynamic evolution among multiple elements within a system, aptly explaining how the five dimensions of teachers’ digital literacy constitute a dynamic ecosystem. Any change in one dimension within the system can influence the overall form and effectiveness of teachers’ digital practices, which in turn affects the classroom atmosphere, teacher-student interactions, and learning support. Ultimately, this has a profound and systematic impact on students’ psychological safety, sense of belonging, and growth experiences, collectively referred to as student happiness.

**Figure 1:**
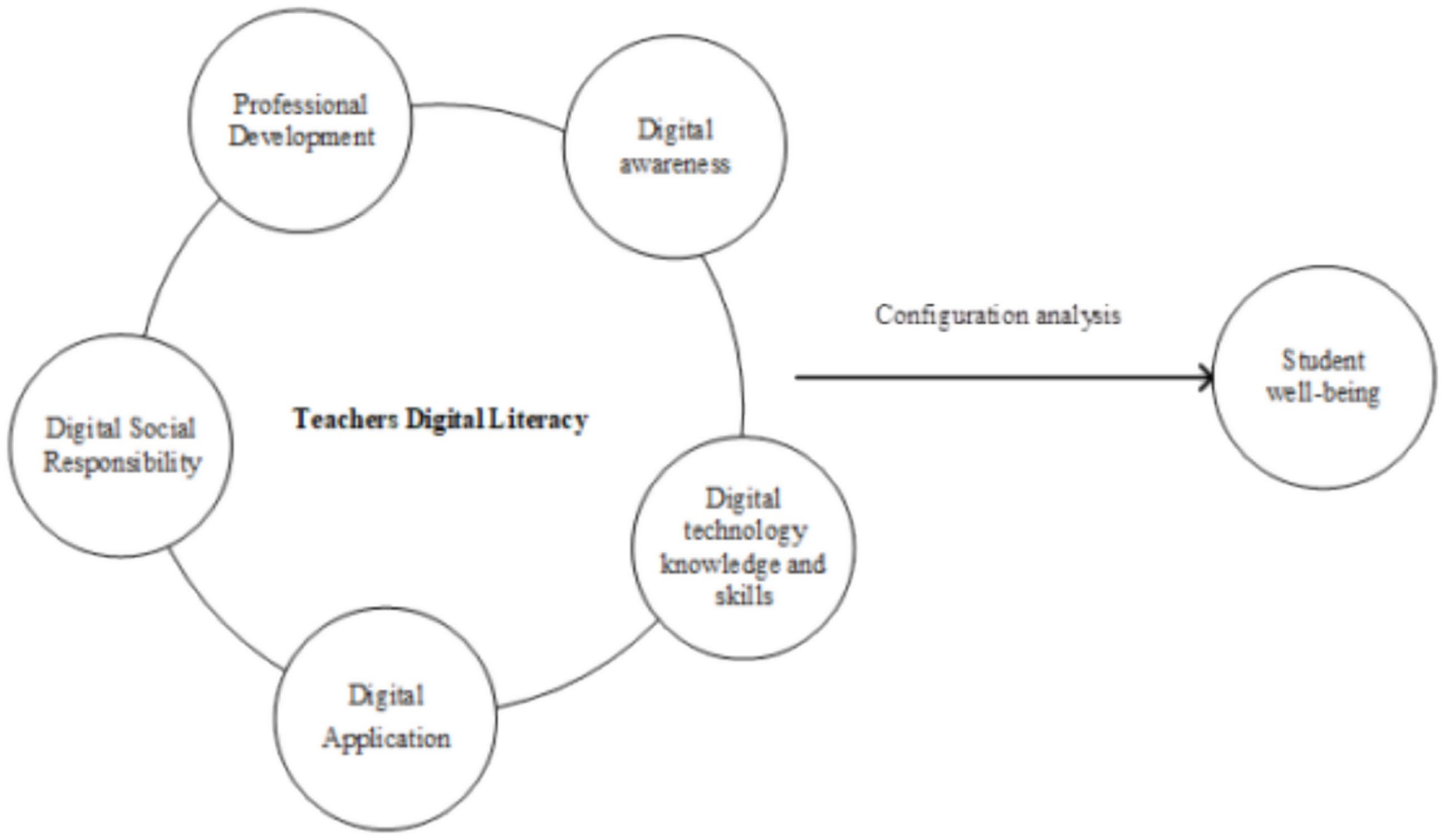
Research model.

From a configurational perspective, the influence of antecedent variables on outcomes is multiple concurrent and asymmetric variables. The configurational perspective and Qualitative Comparative Analysis (QCA) adopt a holistic analytical approach, viewing research subjects as configurations composed of different combinations of condition variables and integrating the advantages of both case studies and variable research. This study employs the five dimensions established by the Chinese Ministry of Education as antecedent conditions to examine teachers’ digital literacy and analyze its impact on student well-being. The influence of the five dimensions of teacher-quality literacy on educational quality and student well-being is complex. These dimensions are interdependent and mutually reinforcing, thus forming a dynamic ecosystem. This multiple concurrent influence mechanism reflects the comprehensive and systemic nature of teachers’ development. At the same time, the relationship between these dimensions and student well-being outcomes is not a simple linear cause-and-effect relationship; instead, it exhibits characteristics of causal asymmetry. This means that the same dimension may yield different educational effects in different contexts, emphasizing the complexity of educational environments and flexibility of teacher behavior. Therefore, adopting a holistic research framework that includes these five dimensions and conducting configurational studies through fuzzy-set Qualitative Comparative Analysis (fsQCA) allows for a comprehensive exploration of how teacher-quality literacy synergistically affects student well-being. This research approach emphasizes the interactions and combined effects among these dimensions, providing a suitable analytical tool for understanding the complex relationship between teacher quality literacy and student well-being. Figure 1 presents the research framework of this study.

## 3 Research design

### 3.1 Research methods

Given the complexity of the composition and functioning mechanisms of teachers’ digital literacy, its impact on students’ well-being is not driven by a single factor; relying solely on single-factor analysis makes it difficult to fully reveal the causal relationship between the two (41). Therefore, to accurately analyze the sufficient conditions and causal configuration effects of different dimensions of teachers’ digital literacy on students’ well-being, this study adopts fuzzy-set Qualitative Comparative Analysis (fsQCA 3.0) to conduct an empirical investigation, exploring the synergistic pathways through which various dimensions of teachers’ digital literacy enhance students’ well-being. The core reason for choosing fsQCA rather than traditional methods such as regression analysis or single-case analysis lies in its key features of multiple concurrency, equifinality, and asymmetry, which align well with this study’s need to analyze “multifactor configurational effects on outcomes.” This method not only overcomes the limitations of single-variable analysis but also identifies different configurations of teachers’ digital literacy that lead to the same outcome—namely, enhanced student well-being—thereby addressing the central research question of this study: “Which combinations of teachers’ digital literacy can effectively enhance students’ well-being?” Moreover, the fuzzy-set calibration characteristics of fsQCA enable better handling of measurement data for latent variables such as teachers’ digital literacy and students’ well-being, making it more consistent with the continuity and fuzziness inherent in variables in educational research. Based on the analytical results of this method, the study can further clarify directions for optimizing teachers’ digital literacy to enhance students’ well-being, providing targeted practical references for promoting high-quality education implementation and strengthening student welfare.

### 3.2 Sample and data

This study adopted a multi-province stratified convenience sampling method. Relying on the public online questionnaire platform Wenjuanxing, an online survey was distributed to university students in higher education institutions across four provinces—Zhejiang, Jiangxi, Jiangsu, and Anhui. By asking respondents to conduct subjective evaluations across five predefined dimensions of teachers’ digital literacy, the data required for the study were collected. During the sampling process, the four provinces in East China were selected as the survey scope, which not only took into account the gradient of educational development levels within the region but also, to a certain extent, reflected the general situation of universities in the eastern and central adjoining regions of China, providing a basis for the regional reference value of the research conclusions. After the questionnaires were distributed, to strictly control data validity, this study established clear screening criteria and conducted data cleaning procedures: invalid questionnaires with highly similar responses or abnormal completion times, including those that were excessively short or long and deviated from a reasonable completion time range, were eliminated. After multiple rounds of verification, a total of 540 valid questionnaires were ultimately retained.

The descriptive statistical characteristics of the sample in this survey are as follows: in terms of gender distribution, there were 306 females (56.7%) and 234 males (43.3%), with the proportion of females slightly higher than that of males, which is consistent with the overall gender distribution of university students; regarding grade distribution, sophomores accounted for the highest proportion (173 students, 32.0%), followed by freshmen (158 students, 29.3%), while juniors (98 students, 18.2%) and seniors (63 students, 11.6%) decreased sequentially, and postgraduate students accounted for the smallest proportion (48 students, 8.9%). The sample covered all undergraduate years as well as postgraduate students, aligning with differences in learning experiences across various stages of higher education; in terms of school geographic location, there were 185 respondents from Zhejiang Province (34.2%), 145 from Jiangsu Province (26.9%), 117 from Jiangxi Province (21.7%), and 93 from Anhui Province (17.2%). The sample distribution across the four provinces was relatively balanced, with no single province accounting for an excessively high proportion.

Overall, the sample in this study achieved multidimensional coverage in terms of gender, grade, and region, and the sample structure is generally consistent with the overall characteristics of university students in the surveyed regions. It demonstrates a certain degree of group representativeness and regional representativeness, effectively reflecting university students’ multidimensional evaluations of teachers’ digital literacy in the four East China provinces, and providing a reliable data foundation for subsequent research analysis.

### 3.3 Variable measurement

The variable measurements in this paper are entirely based on the “Teacher Digital Literacy” framework developed by the Chinese Ministry of Education, which represents a well-established research foundation. All questionnaire scales used the Likert scale method, where a response of “1” indicates a very low degree and “5” indicates a very high degree.

#### 3.3.1 Outcome variables

The outcome variable examined in this study is students’ level of well-being. The study draws on the student well-being analysis framework (Framework for the Analysis of Student Well-being) used by the Organisation for Economic Co-operation and Development (OECD) in the Program for International Student Assessment (PISA) (12). PISA (58) defines well-being as the psychological, cognitive, social, and physical functions and capacities that students need to live happy and fulfilling lives (42). PISA (59) further divides the measurement of students’ psychological well-being into five dimensions: overall life satisfaction, meaning in life, emotional experiences (positive and negative), self-efficacy, and fear of failure (43). Based on the studies of Zheng Xiaoming et al. and Shen Xuejun et al., this paper measures student well-being from two dimensions: learning well-being and psychological well-being (44).

#### 3.3.2 Conditional variables

The conditional variables in this study are the five dimensions of Teachers’ Digital Literacy formulated by the Chinese Ministry of Education. With reference to the Teachers’ Digital Literacy framework, and considering differences in overall faculty quality, equipment levels across universities, and data availability, this study selects the secondary indicators “digital awareness,” “digital willingness,” and “digital volition” to measure teachers’ digital awareness; “digital technology skills” and “digital technology knowledge” to measure digital technology knowledge and skills; “digital teaching design,” “digital teaching implementation,” “digital academic evaluation,” and “digital collaborative education” to measure digital application; the secondary indicators “legal and ethical norms” and “digital security protection” to measure digital social responsibility; and “digital learning and professional development” and “digital teaching research and innovation” to measure professional development. See Table 2 for details.

**Table 2:** Variable selection and measurement.

Variable	Dimension	Measurement indicators
Level of teachers’ digital literacy	Digital Awareness (DA)	Digital Understanding
		Digital Willingness
		Digital Will
	Digital Technology Knowledge and Skills(DTKS)	Digital Technology Skills
		Digital Technology Knowledge
	Digital Application(DP)	Digital Teaching Design
		Implementation of Digital Teaching
		Digital Academic Evaluation
		Digital Collaborative Education
	Digital Social Responsibility (DSR)	Rule of law and ethical standards
		Digital security protection
	Professional development (PD)	Digital learning and research
		Digital Teaching Research and Innovation
Student well-being	Learning Well-Being (LWB)	Campus Classroom Learning Satisfaction
	Psychological Well-being (PWB)	Teaching evaluations of teachers’ digital literacy levels

### 3.4 Reliability and validity testing

This study used SPSS 27.0 statistical analysis software to test the reliability and validity of the Teacher Digital Literacy Scale and the Student Well-being Scale. The test results are shown in Table 3. The study conducted examinations from four dimensions—reliability, content validity, convergent validity, and discriminant validity—to comprehensively assess the reliability and validity of the scales.

**Table 3:** Reliability and validity analysis of teacher digital literacy and student well-being scales.

Variables	Items	Factor loadings	αValue	CR	AVE	Square root of AVE
Digital awareness (DA)	DA1 Teachers can understand the value of digital technology in economic, social, and educational development.	0.806	0.828	0.909	0.666	0.816
	DA2 Teachers can recognize the opportunities and challenges brought by the development of digital technology to teaching and education.	0.871				
	DA3 Teachers have the willingness to actively learn and utilize digital technology resources.	0.864				
	DA4 Teachers possess the initiative to engage in educational digitalization practices, exploration, and innovation	0.719				
	DA5 teachers have the confidence and determination to overcome difficulties and challenges encountered in the practice of educational digitization.	0.812				
Digital technology knowledge and skills (DTKS)	DTKS1 teachers master the concepts and basic principles of common digital technologies	0.846	0.853	0.853	0.660	0.813
	DTKS2 teachers have the ability to select strategies for digital technology resources	0.749				
	DTKS3 teachers’ proficiency in using digital technology resources	0.839				
Digital application (DP)	DP1 Teachers possess the ability to analyze learning situations	0.822	0.964	0.958	0.657	0.811
	DP2 Teachers possess the ability to acquire, manage, and produce digital educational resources	0.773				
	DP3 Teachers can design digital teaching activities	0.706				
	DP4 Teachers can create blended learning environments	0.829				
	DP5 Teachers can utilize digital technology resources to support the organization and management of teaching activities	0.847				
	DP6 Teachers can use digital technology resources to optimize the teaching process	0.812				
	DP7 Teachers can employ digital technology resources to conduct individualized guidance	0.825				
	DP8 Teachers can select and apply evaluation data collection tools	0.804				
	DP9 Teachers can apply data analysis models to analyze academic data.	0.799				
	DP10 Teachers can visualize and interpret academic data.	0.822				
	DP11 Teachers’ level of cultivation of students’ digital literacy.	0.832				
	DP12 Teachers’ moral education abilities developed through the use of digital technology resources	0.849				
Digital social responsibility (DSR)	DSR1 Teachers can enhance students’ awareness of standardizing internet usage in accordance with laws	0.768	0.923	0.921	0.661	0.813
	DSR2 Teachers can guide students to reasonably use digital products and services	0.785				
	DSR3 Teachers can strengthen students’ awareness of maintaining a positive and healthy online environment	0.820				
	DSR4 Teachers can enhance students’ awareness of protecting personal information and privacy	0.816				
	DSR5 Teachers can maintain the security of work data.	0.839				
	DSR6 Teachers will focus on network security protection.	0.846				
Professional development (PD)	PD1 Teachers’ ongoing learning ability using digital technology resources	0.814	0.881	0.877	0.589	0.767
	PD2 Teachers’ ability to use digital technology resources to support reflection and improvement	0.780				
	PD3 Teachers can participate in or lead online training	0.784				
	PD4 Teachers’ ability to conduct digital teaching research	0.730				
	PD5 Teachers’ Ability to Innovate Teaching Modes and Learning Methods	0.725				
Learning well-being (LWB)	LWB1 My learning experience is very enjoyable.	0.780	0.925	0.926	0.676	0.822
	LWB2 Overall, I am very satisfied with what I am learning.	0.781				
	LWB3 I always find ways to enrich my studies.	0.848				
	LWB4 I am generally satisfied with the specific content of my studies.	0.862				
	LWB5 For me, higher education would be a very meaningful experience.	0.821				
	LWB6 I am generally satisfied with the sense of accomplishment I gain from my current studies.	0.837				
Psychological well-being (PWB)	PWB1 In general, I have a positive self-view and feel confident in myself.	0.887	0.791	0.916	0.645	0.803
	PWB2 I really enjoy having in-depth conversations with classmates or friends to get to know each other better.	0.827				
	PWB3I handle many daily tasks and matters quite well.	0.830				
	PWB4 I am considered to be willing to contribute and eager to share my time with others.	0.787				
	PWB5 I am good at managing my time flexibly to complete all my studies.	0.743				
	PWB6 I feel that I have grown a lot as time has passed.	0.734				

#### 3.4.1 Reliability test

This study adopts Cronbach’s *α* coefficient and composite reliability (CR) as reliability testing indicators. Cronbach’s α coefficient is used to measure the internal consistency of the scale, while composite reliability (CR) is used to examine the reliability level of latent variables. Generally, a Cronbach’s α coefficient greater than 0.7 and a CR value greater than 0.8 indicate good scale reliability. The test results show that the Cronbach’s α coefficients of all variables range from 0.791 to 0.964, all exceeding the critical value of 0.7; the composite reliability (CR) values range from 0.804 to 0.958, all higher than the standard value of 0.8. Among them, the psychological well-being dimension has the lowest Cronbach’s α coefficient (0.791) and CR value (0.804), which still meet the reliability testing requirements. These results indicate that the scales used in this study have good internal consistency, a high level of latent variable reliability, and overall scale reliability that meets the standards of empirical research.

#### 3.4.2 Validity test

In terms of content validity, all items in the scales used in this study have clear theoretical and practical foundations. The items of the Teacher Digital Literacy Scale are derived from the official standard framework *Teacher Digital Literacy* formulated by the Ministry of Education; the items of the Student Well-Being Scale are based on the OECD-PISA student well-being analytical framework and have been optimized in combination with relevant domestic research findings. All items have undergone theoretical review and practical screening to ensure that the scales demonstrate good content validity.

In terms of convergent validity, this study selected factor loadings and the average variance extracted (AVE) as indicators for assessing convergent validity. Generally, item factor loadings greater than 0.7 and AVE values greater than 0.5 are required, indicating that latent variables can effectively explain the variance of their observed variables and that convergent validity is satisfactory. The results of exploratory factor analysis show that the factor loadings of all items range from 0.706 to 0.887, all exceeding the critical value of 0.7, and no cross-loadings are observed, indicating strong associations between each item and its corresponding latent variable. The AVE values of all variables range from 0.570 to 0.676, all higher than the standard value of 0.5. Among them, the AVE value of the psychological well-being dimension (0.570) is the lowest, yet it still meets the requirement for convergent validity. These results indicate that the scales used in this study exhibit good convergent validity, and that each observed variable can effectively reflect the core connotation of its corresponding latent variable.

In terms of discriminant validity, this study adopted the square root of AVE method to test discriminant validity by comparing the square root of each latent variable’s AVE with its Pearson correlation coefficients with other variables. If the square root of a latent variable’s AVE is greater than its correlations with other variables, the scale is considered to have good discriminant validity. The results show that the square roots of the AVE values for all variables range from 0.685 to 0.822, all of which are greater than their Pearson correlation coefficients with other latent variables, indicating a high degree of distinction among latent variables and good discriminant validity of the scale.

In summary, the Teacher Digital Literacy Scale and the Student Well-Being Scale used in this study meet the standard requirements for empirical research in terms of reliability and validity across all dimensions. Overall, the scales demonstrate good reliability and validity and are suitable for subsequent empirical analysis.

### 3.5 Data calibration

In In fuzzy-set qualitative comparative analysis (fsQCA), calibration is a core preprocessing step. Its essence lies in transforming raw data into fuzzy-set membership scores based on substantive differences between cases and their theoretical relevance. The calibrated set membership scores are constrained to the interval [0,1], where 1 indicates full membership, 0 indicates full non-membership, and values in between reflect partial membership of cases in the set. This process provides the data foundation for subsequent truth table construction, necessary condition analysis, and configurational path exploration.

Following the standardized analytical procedure of fsQCA, this study systematically reviews the theoretical frameworks and empirical calibration paradigms in existing research and, in light of the data characteristics of the antecedent conditions and outcome variable in this study (all measured on five-point Likert scales), adopts the direct calibration method to perform fuzzy-set transformation (45). The setting of calibration standards strictly adheres to the principle of combining theory-driven and case-oriented approaches. Drawing on the three-value calibration method proposed by Fiss (46), three key thresholds—“full membership,” “crossover point,” and “full non-membership”—are defined to achieve the transformation of raw data into fuzzy-set membership scores.

Considering the theoretical connotations of the variables and the characteristics of the sample distribution in this study, the calibration thresholds for all antecedent conditions and the outcome variable are determined as follows: the full membership threshold (X1) is set at 5.0000, corresponding to the theoretically optimal level of the variable; the crossover point (X2) is set at 3.0000, corresponding to a moderate level of the variable, at which membership and non-membership are equal; and the full non-membership threshold (X3) is set at 1.0000, corresponding to the theoretically lowest level of the variable. The descriptive statistics and specific calibration information for each variable are shown in Table 4. As shown in Table 4, taking digital awareness (DA) as an example, its sample values range from 1.0000 to 5.0000, with a mean of 4.1011 and a standard deviation of 0.8907, which conforms to the distribution logic of the calibration thresholds: cases with values ≥5.0000 are defined as having “full membership” in the set of high digital awareness, cases with values ≤1.0000 are defined as having “full non-membership” in this set, and cases with values = 3.0000 represent the boundary between membership and non-membership. The calibration logic for the remaining antecedent conditions and the outcome variable is consistent with that of digital awareness (DA) and is therefore not elaborated further.

**Table 4:** Descriptive statistics and calibration of outcome variables and condition variables.

Variables	Descriptive analysis				Fuzzy set calibration		
	Mean	Standard deviation	Minimum	Maximum	Full membership	Intersection point	Not affiliated at all
DA	4.1011	0.8907	1.0000	5.0000	5.0000	4.4000	3.4000
DTKS	4.0117	0.9120				4.1333	3.0000
DP	3.9990	0.9099				4.1428	3.2857
DSR	4.0664	0.8716				4.3333	3.5000
PD	3.9826	0.9036				4.2000	3.4000
LWB	3.9358	0.8688				4.0000	3.1666
PWB	3.9340	0.8561				4.1666	3.3333

## 4 Data analysis and empirical results

### 4.1 Necessity analysis of antecedent conditions

In fuzzy-set Qualitative Comparative Analysis (fsQCA), necessity analysis is a core preliminary step in configurational analysis. Its theoretical foundation lies in the asymmetry of causal relationships, meaning that the necessary conditions leading to the presence and absence of a given outcome may differ substantially. The primary purpose of this test is to determine whether a single antecedent condition constitutes a necessary condition for the occurrence of the outcome variable or its negation, thereby providing a basis for condition selection in subsequent sufficiency analysis. The key criterion for necessity analysis is the level of consistency, with a widely accepted threshold of 0.9 in the literature (45): if the consistency level of an antecedent condition is ≥0.9, it indicates that the condition is highly necessary for explaining the outcome and should be given particular attention in the configuration; if the consistency level is <0.9, the condition is not a necessary prerequisite for the occurrence of the outcome, and no causal relationship exists in which a single condition determines the outcome. Focusing on the two dimensions that jointly constitute student well-being as the outcome variable and their negations, this study conducts necessity tests for all antecedent conditions. The results are shown in Tables 5, 6.

**Table 5:** Necessity analysis of single factors for learning well-being (LWB).

Conditions tested	Single factor	LWB		~LWB	
		Consistency	Coverage	Consistency	Coverage
Digital awareness	DA	0.7077	0.7792	0.3704	0.4640
	~DA	0.5132	0.4174	0.8237	0.7623
Digital technology knowledge and skills	DTKS	0.7759	0.7486	0.4361	0.4788
	~DTKS	0.4598	0.4175	0.7710	0.7965
Digital applications	DP	0.7691	0.7928	0.3870	0.4539
	~DP	0.4702	0.4026	0.8234	0.8023
Digital social responsibility	DSR	0.7101	0.7991	0.3390	0.4342
	~DSR	0.4972	0.3980	0.8432	0.7679
Professional development	PD	0.7615	0.8441	0.3342	0.4216
	~PD	0.4781	0.3869	0.8764	0.8070

**Table 6:** Necessity analysis of the single factor for psychological well-being (PWB).

Conditions tested	Single factor	PWB		~PWB	
		Consistency	Coverage	Consistency	Coverage
Digital awareness	DA	0.7891	0.7255	0.3809	0.5463
	~DA	0.5066	0.3440	0.8086	0.8568
Digital technology knowledge and skills	DTKS	0.8220	0.6622	0.4479	0.5630
	~DTKS	0.4576	0.3469	0.7313	0.8650
Digital applications	DP	0.8182	0.7043	0.4058	0.5450
	~DP	0.4714	0.3371	0.7798	0.8700
Digital social responsibility	DSR	0.7659	0.7198	0.3564	0.5226
	~DSR	0.4921	0.3289	0.8089	0.8436
Professional development	PD	0.7900	0.7312	0.3662	0.5288
	~PD	0.4910	0.3318	0.8139	0.8581

As shown by the results in Tables 5, 6: among all antecedent conditions influencing the outcome variable LWB, the single condition PD exhibits the highest consistency level (0.822390); among the antecedent conditions influencing ~LWB, ~PD has the highest consistency level (0.835694); among the antecedent conditions influencing PWB, PD has the highest consistency level (0.807526); and among the antecedent conditions influencing ~PWB, ~PD and ~DP show the highest consistency levels (both 0.821566). Notably, none of the antecedent conditions reach the critical threshold of 0.9 for consistency, indicating that in this study no single antecedent condition can independently constitute a necessary condition for the occurrence of student well-being (LWB, PWB) or its negations (~LWB, ~PWB).

This result essentially reflects the complexity of the mechanisms influencing student well-being: the formation and absence of student well-being are not determined independently by any single factor, but rather arise from complex interactions, synergies, and combinational effects among multiple antecedent conditions, with the independent explanatory and predictive power of any single condition being relatively limited. From a research logic perspective, this finding also provides a sound rationale for subsequent sufficiency analysis and the exploration of configurational pathways—because there are no necessary conditions that need to be excluded, the study can conduct configurational analysis based on all antecedent conditions, thereby systematically revealing the differentiated pathways through which different condition combinations influence student well-being.

### 4.2 Condition set analysis

In accordance with the standardized analytical procedure of fuzzy-set Qualitative Comparative Analysis (fsQCA), this study employed fsQCA 3.0 software to construct a Boolean algebra truth table based on calibrated data from 31 sample cases and to conduct standardized configurational analysis. By identifying effective condition configurations, the study reveals the causal complexity of the mechanisms influencing student well-being. The total sample size of this study was 540, meeting the applicability standards for large-sample QCA research. The 31 samples selected after calibration served as the core analytical cases, providing effective data support for the configurational analysis.

Taking into account the research design, data characteristics, and widely accepted academic standards, this study established the key threshold settings for truth table analysis, with all decisions grounded in explicit justifications and free from subjective determination (47). The consistency threshold was set at 0.80, which is the commonly accepted core standard for sufficiency testing in fsQCA configurational analysis and aligns with mainstream practices in QCA research in the fields of public health and education. This threshold ensures that the configurations included in the analysis possess sufficient explanatory power for the outcome, thereby guaranteeing the rigor of configurational sufficiency. The case frequency threshold was set at 3. Considering the characteristics of potential condition combinations generated by five antecedent conditions, and in reference to frequency-setting principles for medium-sized analytical samples, this threshold ensures that the configurations included in the analysis are supported by an adequate number of cases, avoiding interference from accidental configurations. Empirical calculations further indicate that configurations meeting this frequency threshold cover at least 75% of the core analytical cases, satisfying the sample coverage requirements of fsQCA research (45).

In the derivation and presentation of configurational solutions, this study clearly specified the rules for handling logical remainders and the criteria for distinguishing core and peripheral conditions, standardizing operational details to enhance research replicability. Regarding the treatment of logical remainders, only those highly relevant to the theoretical framework and research constructs of this study were incorporated into the configurational analysis, while irrelevant empty configurations were excluded to prevent the generation of spurious configurations caused by overinterpretation and to ensure the relevance of the solutions to the research theme. With respect to solution selection and condition identification, the results primarily present the intermediate and parsimonious solutions, with the intermediate solution serving as the main basis for interpretation. This is because the intermediate solution balances theoretical plausibility and empirical evidence, avoiding both the oversimplification of parsimonious solutions and the redundancy of complex solutions. Core and peripheral conditions were identified in strict accordance with standardized criteria: conditions appearing in both the intermediate and parsimonious solutions were defined as core conditions, whereas those appearing only in the intermediate solution were defined as peripheral conditions. Regarding configurational notation, the standardized symbolic system of fsQCA research was adopted: a filled circle (●) indicates the presence of a condition, a crossed circle (⊗) indicates the absence of a condition, and a blank indicates that the presence or absence of the condition is irrelevant. Large circles represent core conditions, while small circles represent peripheral conditions. The symbolic notation is consistent with the output specifications of fsQCA 3.0 software.

Based on the above settings and operational norms, this study completed the full analytical process using fsQCA 3.0 software and identified configurations of high and low well-being corresponding to learning well-being and psychological well-being, respectively. All analytical procedures strictly followed the standardized workflow of the software. The specific results for the condition composition, core/peripheral attributes, and related indicators of each configuration are presented in Table 7. Subsequently, this study will conduct robustness tests in accordance with fsQCA research standards to verify the reliability of the configurational analysis results, and the relevant test results will be reported in detail in conjunction with the actual analysis.

**Table 7:** High well-being configurations / non-high well-being configurations.

Configuration	High sense of happiness			Non-high sense of happiness							
	LWB		PWB	~LWB				~PWB			
	S1a	S1b	S1c	NS1a	NS1b	NS1c	NS1d	NS1e	NS1f	NS1g	NS1h
DA	●		●		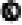	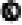	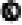	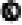			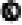
DTKS		●	●	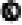		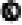	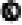	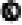		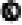	
DP	●	●	●	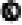	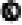		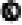		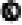	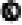	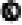
DSR	●	●	●	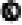	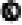	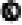			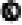	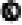	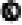
PD	●	●	●	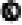	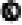	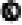	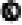		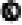		
Consistency	0.893	0.890	0.855	0.892	0.876	0.898	0.898	0.905	0.910	0.923	0.920
Raw-coverage	0.552	0.590	0.604	0.696	0.716	0.678	0.683	0.675	0.710	0.656	0.682
Unique coverage	0.024	0.061	0.604	0.003	0.005	0.002	0.016	0.005	0.019	0.007	0.006
Solution consistency	0.888		0.855	0.863				0.880			
Solution coverage	0.614		0.604	0.772				0.791			

● core condition present, core condition absent, 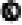 peripheral condition present, peripheral condition absent, “space” indicates that the condition may or may not be present.

### 4.3 Robustness test

To assess the robustness of the configurational analysis results regarding the antecedents of teachers’ digital literacy and to examine their sensitivity to key analytical parameter settings, this study systematically conducted two robustness checks. The first check focused on the case frequency threshold. Based on the baseline analysis, the case frequency threshold was relaxed from 3 to 2 for reanalysis. The results are shown in Table 8. The core findings remained stable: the three configurations (S2a, S2b, S2c) leading to high student well-being—namely, the pathways in which all five dimensions of teachers’ digital literacy (DA, DTKS, DP, DSR, PD) are present—were completely consistent with the baseline analysis in Table 7 in terms of both conditional composition and core indicators. This indicates that the core positive pathways are not affected by whether a small number of marginal cases are included, demonstrating a solid foundation.

**Table 8:** Robustness test results for high well-being configurations / non-high well-being configurations 1.

Configuration	High Well-being			Low subjective well-being							
	LWB		PWB	~LWB			~PWB				
	S2a	S2b	S2c	NS2a	NS2b	NS2C	NS2d	NS2e	NS2f	NS2g	NS2h
DA	●		●	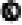		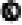		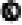	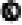		
DTKS		●	●		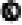	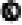			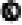	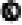	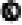
DP	●	●	●	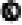	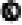		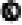	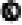			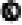
DSR	●	●	●		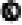	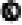				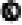	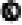
PD	●	●	●	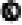	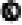	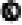	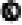			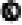	
Consistency	0.893	0.890	0.855	0.867	0.892	0.898	0.899	0.904	0.907	0.924	0.923
Raw-coverage	0.552	0.590	0.604	0.736	0.696	0.678	0.734	0.706	0.675	0.650	0.656
Unique coverage	0.024	0.061	0.604	0.007	0.003	0.001	0.002	0.009	0.003	0.003	0.007
Solution consistency	0.888		0.855	0.857			0.869				
Solution coverage	0.614		0.604	0.777			0.807				

● core condition present, core condition absent, 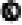 peripheral condition present, peripheral condition absent, “space” indicates that the condition may or may not be present.

The second check targeted the criterion of statistical sufficiency. With the case frequency threshold restored to 3, the consistency threshold was raised from 0.8 to a more stringent 0.85 for reanalysis. The results are shown in Table 9. This test yielded deeper theoretical insights. First, the three core pathways leading to high well-being (S3a–c) were perfectly replicated once again, providing dual validation of their robustness. Second, the interpretation of non–high well-being pathways was optimized, as the stricter criterion filtered out the “all teacher competencies absent” configuration from the baseline model. The adjusted model more clearly reveals that the robust pathways leading to non–high well-being are generally characterized by the absence of two foundational competencies—digital awareness (DA) and digital technology knowledge and skills (DTKS). Meanwhile, the overall solution consistency and coverage remained above the ideal levels of 0.84 and 0.79, respectively, after adjustment, indicating that the research conclusions are reliable. In summary, by systematically varying the criteria for case inclusion and statistical sufficiency, the core pathways identified in this study have successfully passed rigorous robustness tests.

**Table 9:** Robustness test results for high well-being configurations / non-high well-being configurations 2.

Configuration	High Well-being			Low subjective well-being								
	LWB		PWB	~LWB				~PWB				
	S3a	S3b	S3c	NS3a	NS3b	NS3C	NS3d	NS3e	NS3f	NS3g	NS3h	NS3i
DA	●		●			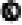	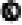			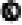	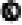	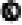
DTKS		●	●	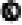	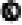				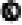			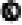
DP	●	●	●		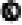		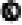	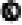	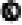		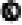	
DSR	●	●	●	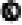		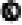				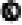		
PD	●	●	●	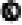	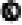	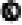	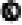	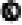				
Consistency	0.893	0.890	0.855	0.882	0.884	0.867	0.867	0.899	0.906	0.905	0.904	0.907
Raw-coverage	0.552	0.590	0.604	0.710	0.713	0.742	0.736	0.734	0.679	0.724	0.706	0.675
Unique coverage	0.024	0.061	0.604	0.002	0.002	0.015	0.004	0.021	0.010	0.025	0.002	0.020
Solution consistency	0.887		0.855	0.843				0.862				
Solution coverage	0.614		0.604	0.795				0.831				

● core condition present, core condition absent, 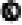 peripheral condition present, peripheral condition absent, “space” indicates that the condition may or may not be present.

## 5 Research conclusion and implications

### 5.1 Research findings

Based on the constructed research model, this study selected five antecedent conditions—professional development, digital application, digital social responsibility, digital awareness, and digital technology knowledge and skills—and adopted the fuzzy-set qualitative comparative analysis (fsQCA) method. Using 540 valid samples from four provinces—Zhejiang, Jiangsu, Jiangxi, and Anhui—as cases, this study systematically explored the multiple concurrent causal relationships between various dimensions of teachers’ digital literacy and students’ well-being. The main findings are as follows:

The enhancement of students’ well-being depends on the multidimensional synergy of teachers’ digital literacy rather than the linear effect of any single factor. Necessity analysis indicates that none of the five literacy dimensions constitutes a necessary condition for high student well-being, corroborating the multifaceted and complex mechanisms influencing adolescent mental health. However, configurational analysis further reveals that professional development (PD), digital application (DP), and digital social responsibility (DSR) are consistently present in high well-being configurations, forming a core cluster of competencies that support student well-being. This suggests that in school health promotion and teacher development, priority should be given to these three universally associated literacy dimensions, treating them as foundational intervention targets for enhancing the psychological well-being of student populations.

There are three equivalent and robust configurational pathways for achieving high student well-being, providing a scientific basis for differentiated interventions. The study finds that teachers’ digital literacy can jointly lead to high well-being through three typical combinations of conditions: Awareness-led type: digital awareness (DA) serves as the value orientation, working in synergy with professional development (PD), digital practice (DP), and digital social responsibility (DSR); Skills-empowerment type: building on the above three elements, digital technology knowledge and skills (DTKS) are further incorporated to provide practical support; System-synergy type: all five literacies are fully coupled to form a comprehensive support system for holistic development. All three pathways remain stable in robustness tests across case frequency thresholds and consistency thresholds. This result indicates that there is no single optimal pathway for enhancing student well-being. Schools can select differentiated strategies—driven by shifts in awareness or grounded in skills development—according to their own resource conditions and stages of development. This approach helps avoid the inefficiencies that may arise from adopting integrated or one-size-fits-all intervention models, thereby enabling more precise allocation of public health resources.

The causes of low happiness exhibit a “critical shortfall” pattern and display marked asymmetry with the pathways to high happiness. The study identifies multiple configurations leading to low happiness, which are more numerous and structurally more complex. Robustness tests further clarify that deficiencies in digital awareness (DA) and digital technology knowledge and skills (DTKS) constitute common “shortfalls” across these negative pathways. This indicates that even when teachers perform well in other competencies, a lack of foundational digital cognition or core skills still makes it difficult to effectively support student well-being. This asymmetry warns that simply replicating positive combinations associated with high well-being cannot automatically eliminate negative outcomes; instead, it is necessary to selectively strengthen key competency gaps, especially by implementing preventive interventions in digital cognition and skills, which should become a priority action within school mental health promotion systems.

In sum, from a configurational perspective, this study reveals the multiple concurrent mechanisms through which teachers’ digital literacy influences student well-being, providing a new analytical framework for understanding the social determinants of adolescent mental health. The conclusions not only underscore the importance of competency synergy but also point to differentiated and precision-oriented pathways for teacher development and key targets for shortfall-focused interventions, offering clear implications for school public health policymaking, the design of mental health promotion programs, and teacher professional development practice.

### 5.2 Theoretical significance

From a configurational theory perspective, this study employs fuzzy-set qualitative comparative analysis (fsQCA) to systematically examine the coupling effects among the various components of teachers’ digital literacy on students’ well-being. The findings not only analyze the complex causal relationships between teachers’ digital literacy and students’ well-being—thereby deepening, refining, and extending existing theories—but also provide multi-level theoretical contributions and practical implications for the fields of public health and education policy.

This study deepens the understanding of the causal complexity among the elements of digital literacy. Existing research has mostly treated the various dimensions of digital literacy as variables with independent and parallel effects on student development (48, 49). By contrast, using fuzzy-set qualitative comparative analysis, this study reveals an asymmetric hierarchy of effects among five categories of literacy elements: career development literacy, digital application literacy, and digital social responsibility constitute the core conditions influencing students’ well-being, while digital awareness and technical knowledge and skills primarily function as auxiliary conditions. This finding moves beyond the simplification paradigm that treats all elements as equally important (50), clarifies the asymmetry and structural interdependence among the elements, and thus advances the theoretical construction of teachers’ professional literacy in the digital age, providing a more precise framework for understanding its mechanisms of influence.

The study reveals the inherent synergistic effects and combinatorial logic of teachers’ digital literacy, providing empirical evidence for a systematic framework for literacy development. The analysis shows that the promotion of students’ well-being is most pronounced when the three core elements are present simultaneously, whereas the absence of digital social responsibility significantly weakens the combined effects of the other elements. This finding theoretically challenges the commonly observed “single-point breakthrough” development pathway in practice and powerfully demonstrates the holistic nature of digital literacy as a synergistic system, whose impact stems from the specific combinations and interactions of key elements rather than the independent effects of individual variables. This offers educational administrators a new theoretical entry point for planning and evaluating the development of teachers’ digital literacy from a systematic and combinatorial perspective, and provides direct theoretical support for education authorities to optimize teacher training programs, echoing the calls and assessment feedback of a considerable number of scholars for the comprehensive enhancement of teachers’ digital literacy levels (50, 51).

This study extends the research dimensions of digital transformation in education and adolescent health promotion. Current research in public health and education largely focuses on the effects of technological applications on academic performance or teaching efficiency (52), while paying insufficient attention to students’ emotional well-being and mental health indicators and generally overlooking the concurrent causal relationships among literacy components. By placing digital social responsibility—an ethical dimension—at the center of analysis, this study not only demonstrates its necessity for enhancing students’ well-being, but also reveals through configurational analysis that even when teachers’ technological competence is relatively weak, strong digital social responsibility, in synergy with other factors, can still effectively promote students’ emotional health. This conclusion extends the scope of research on digital education ethics (53) from individual teacher norms to the public health practice of promoting adolescent mental health, enriches the theoretical framework of population health determinants, and provides critical theoretical support for balancing technological efficiency and humanistic care in the process of digitalization.

The analytical approach and core findings of this study, developed within a specific national context and policy framework, offer significant theoretical insights and reference value for understanding the complex impacts of digital literacy in broader contexts. First, the configurational perspective and fuzzy-set qualitative comparative analysis (fsQCA) method adopted in this research reveal how multiple literacy elements collectively influence student well-being through different combinations. This analytical approach itself provides a transferable research path and tool for exploring similar multifactorial interaction mechanisms across various educational systems with different policy conditions and technological development stages. Second, this study empirically establishes “digital social responsibility” as a core condition affecting student well-being. This conclusion not only highlights the foundational role of the ethical dimension in the digital transformation of education but also offers testable theoretical clues for understanding “how ethics can mitigate technological impacts” in other contexts. It suggests that, even in environments with uneven technological capabilities, prioritizing digital ethics may still be key to safeguarding student welfare. Therefore, the value of this study lies in its clear revelation of the interaction logic between key mechanisms and elements within a specific context, providing solid empirical references and an inspiring analytical framework for future comparative studies, theoretical validation, and expansion across different cultural or institutional backgrounds.

### 5.3 Practical implications

The findings of this study indicate that teacher digital literacy plays a central driving role and exhibits multi-path synergy in promoting student well-being, while also presenting asymmetric risks. This suggests that the education system should focus on both systemic health promotion and targeted risk intervention, using the development of teacher digital literacy as a key lever to effectively enhance student well-being and mental health levels. Based on the core principles of public health—“prevention first, categorized intervention, and multi-level collaboration”—the following three levels are proposed for constructing implementation pathways:

At the individual and teaching practice level, teachers themselves need to proactively and continuously update their digital knowledge and skills and embrace the concept of lifelong learning. While actively planning their personal digital competence development pathways and enhancing their literacy, teachers should particularly regard digital social responsibility as a necessary professional practice for protecting students’ mental health. Firstly, teachers need to internalize digital social responsibility into daily health education and teaching practices. Through direct modeling and explicit instruction, they should actively guide students in responsible digital interactions during the daily use of digital tools to prevent direct harm to mental health from issues such as cyberbullying and privacy breaches. This, in itself, serves as effective primary prevention. Secondly, teachers can leverage their digital application skills to implement precise secondary prevention and early support. For instance, designing curriculum modules based on digital citizenship education, organizing online ethical discussions to help students develop critical awareness and competence in using technology, or utilizing digital tools to create safe and anonymous feedback channels for regular assessments of students’ emotional states, thereby providing timely attention to students with potential needs. In education and teaching, guiding students to correctly understand and use digital technology can gradually cultivate their digital citizenship awareness and ethical judgment.

At the school organization and health management level, as the primary arena for promoting adolescent mental health, schools should comprehensively integrate teacher digital literacy training into the school health promotion framework, implementing strategies that simultaneously solidify core competencies and mitigate health risks. On one hand, schools need to systematically translate professional development, digital application, and particularly digital social responsibility—the three core competencies—into practical skills training for mental health promotion. Examples include workshops on “early psychological risk identification based on learning data” and “peer support guidance in digital environments.” On the other hand, school administrators should adopt risk group management approaches from public health. Specifically, they should develop school-based early warning and intervention mechanisms targeting the various non-high-well-being risk configurations identified in this study, especially those involving the combination of high digital application skills coupled with a lack of digital social responsibility. For instance, specialized training on digital ethics and health communication could be provided for teacher groups at risk of weak digital social responsibility, while teachers with insufficient digital application skills could be supported through mentoring programs to enhance their ability to use technology for emotional communication and support, thereby blocking the transmission of specific risk combinations to student groups.

At the policy and public health governance levels, education and public health departments should collaboratively build a health promotion-oriented policy framework for teacher digital literacy. First, based on the baseline mental health levels of school regions and student groups, differentiated and phased literacy development guidelines can be formulated. For example, in regions or schools with higher emotional health risks among students, policies should prioritize strengthening teachers’ digital social responsibility and digital application capabilities. This ensures that teachers can provide stable emotional support to students through digital tools and effectively guide them in resisting public health risks such as online bullying and information addiction. Second, a health outcome-oriented monitoring and evaluation system should be improved. Core indicators of student well-being and mental health, particularly those related to a sense of security in the digital environment, should be incorporated into the evaluation of the effectiveness of teacher digital literacy development. This will shift policies from mere technical skill assessments to a focus on population health benefits. Furthermore, there is a need to increase the allocation of public health resources toward educational digitization, especially in resource-constrained areas. Priority should be given to building digital infrastructure related to mental health support and training teachers in using these tools for early identification and preliminary intervention, thereby narrowing the “health digital divide” among different groups.

In summary, from a public health perspective, enhancing teacher digital literacy is not only a requirement of educational digital transformation but also a strategic intervention concerning the mental health of young people. It demands concerted efforts from policymakers, school administrators, and frontline teachers. By building on core competencies and implementing classified guidance and risk management, the ultimate goal is to achieve a fundamental shift in the educational ecosystem of the digital age—from improving the efficiency of knowledge transmission to promoting the comprehensive and healthy development of individuals.

### 5.4 Research limitations and future prospects

This study still has several limitations, which provide directions for further research. First, this study employs a cross-sectional design, which presents limitations in causal inference. Although the QCA method effectively reveals the configurational relationships among indicators, it cannot establish causal relationships or temporal directions between variables. The observed association between teachers’ digital literacy and students’ well-being may be influenced by reverse causality or unobserved confounding variables, which also restricts inferences about the long-term effects of teachers’ digital literacy on student well-being. Second, the measurement of teachers’ digital literacy relies entirely on students’ perceptions, which may be affected by social desirability bias or individual differences in student perceptions. Future research could incorporate more objective measurement methods, such as teachers’ self-assessments, classroom observation records, or actual digital skills tests, to obtain more comprehensive and accurate data. Third, student well-being is a complex multi-dimensional construct encompassing affective experiences, academic satisfaction, interpersonal relationships, self-worth, and other aspects. This study has certain limitations at the measurement level (54–56). Finally, this study primarily focuses on individual-level variables and does not adequately incorporate broader institutional or socioeconomic factors (such as variables related to school digital support environments, university digital literacy support policies, and regional economic development levels), which to some extent simplifies the complexity of the real educational ecosystem (57). These factors may interact in complex ways with teachers’ digital literacy and student well-being. Future model construction should consider incorporating these macro- or meso-level variables to achieve a more systematic understanding.

Based on the above limitations, future research could be further deepened and expanded in the following aspects. In terms of advancing causal research design, longitudinal tracking or randomized controlled experimental designs could be adopted to address the causal inference shortcomings of cross-sectional designs, precisely clarifying the causal mechanisms between the development of teachers’ digital literacy and changes in student well-being, and verifying the long-term effects of teachers’ digital literacy on student well-being. In terms of optimizing measurement methods and reducing bias risks, the assessment of teachers’ digital literacy levels should integrate more objective measurement methods, such as teachers’ self-assessments, classroom observation records, or actual digital skills tests, to complement student perceptual evaluations with multi-source data, enhancing the comprehensiveness and accuracy of measurements. In terms of incorporating multi-level variables and improving research validity, future model construction should include institutional and socioeconomic factors such as school digital infrastructure, university support policies, and regional economic differences in configurational analysis, exploring the interactions of these meso- and macro-level variables with teachers’ digital literacy and student well-being. Additionally, it is important to expand samples and deepen measurements by including more diverse regional and school-type samples and employing more comprehensive, multi-source well-being measurement tools.

## Data Availability

The raw data supporting the conclusions of this article will be made available by the authors, without undue reservation.
